# Recent progress in proteins regulating the germination of *Bacillus subtilis* spores

**DOI:** 10.1128/jb.00285-24

**Published:** 2025-01-08

**Authors:** Tianyu Zhang, Ziqi Gong, Bing Zhou, Lei Rao, Xiaojun Liao

**Affiliations:** 1 College of Food Science and Nutritional Engineering, National Engineering Research Center for Fruit and Vegetable Processing, Key Laboratory of Fruit and Vegetable Processing of Ministry of Agriculture and Rural Affairs, Beijing Key Laboratory for Food Non-Thermal Processing, China Agricultural University 630105 , Beijing, China; 2 Shanghai Institute of Immunity and Infection, Chinese Academy of Sciences 85402 , Shanghai, China; University of Massachusetts Chan Medical School, Worcester, Massachusetts, USA

**Keywords:** *Bacillus subtilis*, spore, germination, regulatory proteins, functional mechanism

## Abstract

Bacterial spores can remain dormant for years, but they maintain the ability to recommence life through a process termed germination. Although spore germination has been reviewed many times, recent work has provided novel conceptual and molecular understandings of this important process. By using *Bacillus subtilis* as a model organism, here we thoroughly describe the signal transduction pathway and events that lead to spore germination, incorporating the latest findings on transcription and translation that are likely detected during germination. Then, we comprehensively review the proteins associated with germination and their respective functions. Notably, the typical germinant receptor GerA and the SpoVAF/FigP complex have been newly established as channels for ions release at early stage of germination. Moreover, given that germination is also affected by spore quality, such as molecular cargo, we collect the data about the proteins regulating sporulation to affect spore quality. Specifically, RocG-mediated glutamate catabolism during sporulation to ensure spore quality; GerE-regulated coat protein expression, and CotH-modified coat protein by phosphorylation to ensure normal coat assembly; and RNase Y-degraded RNA in newly released spores to promote dormancy. The latest progress in our understanding of these germination proteins provides valuable insights into the mechanism underlying germination.

## INTRODUCTION

Bacteria in orders of *Bacillales* and *Clostridiales* can undergo sporulation to form dormant and resistant spores for surviving adverse environments such as nutrient limitation (1). The process of sporulation contains several physiological events mainly including (2–4): (i) asymmetric cell division to form a larger mother cell and a smaller forespore; (ii) engulfment of the forespore by the mother cell; (iii) forespore maturation accomplished by cortex formation between the inner and outer membrane, coat assembly on the out membrane, and the uptake of a large amount of pyridine-2, 6-dicarboxylic acid (DPA) mainly chelated to divalent Ca^2+^ ions (Ca-DPA) via SpoVA protein channel; and (iv) lysis of the mother cell to release the mature and resistant spore. The uptake of Ca-DPA and cortex formation play crucial roles in reducing the core water content to facilitate spore maturation (5, 6), but the underlying mechanisms are unclear. The mature spores are in a metabolically dormant state and exhibit extremely high resistance to a variety of external insults, including heat, chemicals, UV radiation, and desiccation (5–7). For detailed knowledge about spore resistance, the readers can refer to the elegant and comprehensive reviews (5–11).

The high resistance of spores enables them to be widespread in diverse natural environments, inevitably impacting human life (7, 12). Several spore-forming bacterial species, including *Bacillus cereus*, *Clostridium botulinum*, *Clostridium perfringens*, *Clostridium difficile*, and *Bacillus anthracis*, are known for their pathogenic potential, threatening human health (13–17). Furthermore, the spores can survive different food sterilization processes, causing food spoilage and economic loss (1, 17, 18). Although these spore-forming bacteria are harmful, it is crucial to recognize that they primarily exist in the environment as dormant spores, which are non-pathogenic. Only upon germination and subsequent outgrowth into vegetative cells do they resume cellular activity and begin to synthesize toxins or other virulence factors. Consequently, understanding the mechanisms of germination, the first and critical event for spore revival, has emerged as a key area of research (19–22).

Physiological germination of spores of *Bacillus* species typically begins with the interaction of germinants (such as sugars, amino acids, or purine nucleosides) with their respective germination receptors (GRs), which are exclusively produced in the forespore and inserted into the spore inner membrane (IM) (20, 23, 24). Once the GRs are activated, the spores will irreversibly proceed through subsequent germination events, mainly including the release of cations and Ca-DPA, and then cortex hydrolysis, leading to the full hydration of the spore core and the completion of germination (20, 25). During this process, the spore’s morphology shifts from phase-bright to phase-gray to phase-dark (25). *B. subtilis*, owing to its fully sequenced genome and the ease of genetic manipulation, is widely utilized as a model organism in spore germination studies. Also, the knowledge of spore germination acquired from *B. subtilis* is largely applicable to other species from the *Bacillus* genus (19, 20).

The highly organized and sequential events of spore germination are exquisitely regulated by a variety of proteins. Typically, GRs sense external germination signals, SpoVA proteins form channels responsible for Ca-DPA release, and cortex lytic enzymes (CLEs) facilitate cortex degradation (19, 20, 25). These proteins are directly essential for germination. Additionally, some factors, such as the transcriptional factor GerE and the glutamate dehydrogenase RocG, can affect the sporulation process and thus indirectly regulate germination (26, 27). A comprehensive understanding of the proteins involved in germination would facilitate the development of effective spore control methods in food and medical fields. In this review, we present an updated list of proteins associated with *B. subtilis* spore germination, including those involved in the germination pathway and the ones regulating sporulation to affect germination. Furthermore, we elucidate the mechanisms by which these proteins regulate or influence germination, based on the latest research evidence.

## GERMINATION OF A *B. SUBTILIS* SPORE AND RELATED PROTEINS

**Fig 1:**
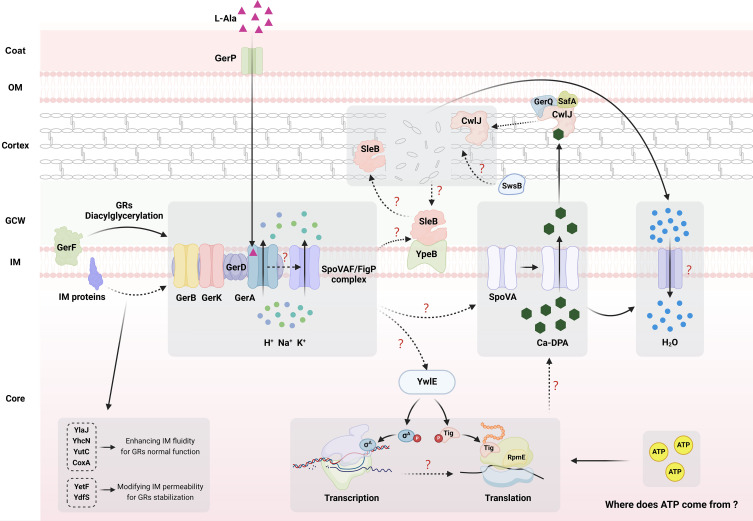
The germination pathway and related proteins in a *Bacillus subtilis* spore. The germination is initiated by the binding of l-Ala to GerA with the assistance of GerP, subsequently inducing several physiological events: (i) l-Ala binds and somehow activates GerA in the IM. GerF contributes to the diacylglycerylation of the C subunits of GRs, and lipoproteins modulate the properties of the IM, ensuring GRs normal function. (ii) The activated GerA releases ions of H^+^, K^+^, and Na^+^ with the assistance of SpoVAF/FigP complex. (3) The ions release somehow triggers the opening of SpoVA channel for substantial Ca-DPA release. At this stage, YwlE-mediated transcription and translation are detected, but their relationships between the upstream ions release or downstream Ca-DPA release are unclear. (iv) Ca-DPA release leads to partial core hydration, and the Ca-DPA molecules further activate CwlJ for cortex hydrolysis, which in turn somehow activates SleB. Meanwhile, the activated GerA is also supposed to be related to SleB activation, and SwsB is proposed to modify the cortex structure for CwlJ cleavage. CwlJ and SleB cooperate to hydrolyze the cortex and promote complete core hydration. The structures in the diagram are not drawn to scale, and question marks and dashed lines represent unknown mechanisms and pathways. CLE, cortex lytic enzyme; GCW, germ cell wall; GR, germination receptor; IM, inner membrane.

The germination process of a *B. subtilis* spore comprises several physiological events, including germinant binding, GR activation, signal transduction, Ca-DPA release, and cortex hydrolysis (20, 21). Although germinants are specifically recognized by GRs, with GerA recognizing l-Alanine (l-Ala), and GerB and GerK recognizing a mixture of l-asparagine, d-glucose, d-fructose, and K^+^ (AGFK) (25), the subsequent events in the germination are remarkably similar (20). Therefore, the well-documented germination process initiated by l-Ala will be employed as the primary model in this review to further elucidate the proteins associated with germination (Fig. 1). The details of individual germination event are then described in the following text.

1. Germinant binding: at the germination initiation stage, it is essential for l-Ala to penetrate the outer layers, including the spore coat, outer membrane and cortex, to reach and bind to the GerA receptor situated on the IM (24, 25, 28). During this process, GerP proteins within the coat facilitate the access of l-Ala to GerA (29).
2. GRs activation (commitment stage): after l-Ala binding to GerA, spores enter the commitment stage, at which the germination process becomes irreversible (30). At this stage, the GerA complex, acting as a nutrient-gated channel, is supposed to undergo a potential conformational change that leads to channel opening (31). This opening results in the release of H^+^, Na^+^, K^+^, and a small amount of Ca-DPA (20, 31). Recent evidence suggests that the SpoVAF/FigP complex can also act as an ion channel, enhancing the germination process (32). Moreover, another ion channel protein YugO has been reported to be involved in K^+^ release during spore germination (33). However, the precise function of ion release by YugO is obscure and controversial (see the below discussion in GerA) (34). Notably, the commitment time for individual spores within a group can vary significantly, ranging from minutes to hours or even days (20), which may partially contribute to the observed germination heterogeneity (20, 35). Variations in GR levels, coat integrity, or metabolite contents among individual spores, potentially resulting from a “bet-hedging” strategy (26, 27, 35, 36), could also play a role in germination heterogeneity. Significantly, at the commitment stage, the spores lose their resistance to innocuous heat or acid treatments (37).
3. Signal transduction and DPA release: subsequently, the germination signal is transferred from GerA to the Ca-DPA channel SpoVA proteins located in the spore IM (38). This signal transduction has been suggested to occur through the physical interaction between GerA and SpoVA proteins (39). However, a recent study has revealed that SpoVA channel from *C. difficile* (only sharing approximately 46% identity with *B. subtilis* SpoVA) can complement the loss of *B. subtilis* SpoVA, which disrupts the typical GerA-SpoVA contact (31). This finding suggests that the activation of SpoVA by GerA-family receptors may not rely on direct protein-protein interactions but rather on some form of chemical or physical alteration within the spore (31). Nonetheless, following the signal transduction, SpoVA channels are opening to release Ca-DPA from the spore core. Simultaneously, a partial core hydration occurs, increasing the water content from about 35% to approximately 45% (20, 40–42). Unlike the variable commitment times, the time for an individual spore to release its Ca-DPA is about 2 min, demonstrating remarkable consistency across spores within a group (20, 40). At this stage, resistance to wet heat is partially reduced, while resistance to H_2_O_2_ is completely lost (41). Ca-DPA release in this stage turns the spore from phase-bright to phase-gray (19, 42). Notably, while the traditional perspective has been that macromolecular synthesis does not resume until later stages of germination, recent work suggests that transcription and translation can be detected in germinating spores even when they are still phase-bright (22, 43–45), that is, at a stage concurrent with or prior to Ca-DPA release. These findings infer that spores may resume cellular activity earlier as presumed, though this point of view is still controversial (see the “Perspective” section) (42, 46–49).
4. Cortex hydrolysis: Ca-DPA release and partial core hydration further induce cortex hydrolysis, a critical step in spore germination executed by the CLE CwlJ and SleB (20, 50), either of which are sufficient for cortex hydrolysis and thus complete spore germination (25). These enzymes localize to the cortex region but are held inactivate until germination is triggered through different mechanisms (20). CwlJ is produced in the mother cell and deposited in the spore coat close to the outer edge of the cortex (51), while SleB is produced in the forespore and localized at the IM near the inner edge of the cortex (52). Accordingly, once activated, CwlJ works from the outside in, and SleB works from the inside out (53). The activation of these enzymes is supposed to be as follows: the release of Ca-DPA activates CwlJ, catalyzing cortex degradation from the outside in (25, 50). In parallel, partial core hydration or CwlJ-induced cortex hydrolysis somehow facilitates the activation of SleB (54). Additionally, it has been proposed that the germination signal from activated GerA receptors can also promote activating SleB to initiate cortex degradation from the inside out (55). The combined actions of CwlJ and SleB degrade the cortex in a bidirectional manner, leading to further hydration of the spore core and an approximately twofold increase in its volume (42). This degradation process takes about 6–15 min, resulting in the water content of the spore core gradually reaching 80% of its wet weight (41, 42, 56, 57). Subsequently, the spore completes its germination, losing most of its resistance and changing from phase-gray to phase-dark (20, 25, 42).

Except for the typical nutrient germinants, the peptidoglycan (PG) fragments from growing cells have also been reported capable of triggering spore germination of *B. subtilis* via binding to Ser/Thr protein kinase PrkC and inducing downstream phosphorylation events (58, 59). However, the exact mechanism underlying this PrkC pathway remains unclear, and there has been very little follow-up research on this pathway since its discovery. Moreover, certain non-nutrient small molecules can alternatively trigger spore germination by activating the intermediate steps of nutrient-induced germination. Specifically, exogenous Ca-DPA activates the CLE CwlJ to hydrolyze the cortex (60), and the surfactant dodecylamine can trigger spore germination by opening the SpoVA protein channel for Ca-DPA release (39, 61). Interestingly, the physical effect of high hydrostatic pressure is also capable of triggering spore germination, with the underlying mechanism associated with the specific level of pressure applied (21, 62). Specifically, a pressure of 50–300 MPa induces germination by activating nutrient GRs (63–67), while pressures above 400 MPa induce germination by opening the SpoVA channels (63, 67, 68).

Collectively, the key proteins that regulate *B. subtilis* spore germination include GerP, for regulating germinant permeability (69), GerA/GerB/GerK for germinant sensing and signal transduction (20, 24, 25, 58), SpoVA for Ca-DPA release (38, 39, 61, 70), and CwlJ/SleB for cortex hydrolysis (50, 71). Additional factors that function to indirectly regulate germination include GerD and the family of spore IM proteins that promote GerA/GerB/GerK assembly and stability (72); GerF, which promotes the acylation of the C subunit of GRs (73, 74); GerQ/SwsB/YpeB, which regulate CLE activation (50, 53, 75); McsB/YwlE/SigA, which regulate transcription in germinating spores (22, 44); and RpmE/Tig, which regulate translation in germinating spores (22, 45). In the following text, we will introduce their functions and mechanisms of action in detail (Fig. 1).

### GerP

GerP subunits are encoded by the hexacistronic *gerP* operon and located in the inner coat of spore (29). They are thought to form the channels that facilitate the penetration of germinants to reach GRs, thereby enhancing the probability of binding (29, 76). In the absence of the *gerP* operon, spore germination triggered by nutrient germinants such as l-Ala, l-Val, or AGFK is hindered, despite no discernible morphological defects in the spore coat or any malfunction in the germination machinery (69). In addition, germination induced by Ca-DPA, which is non-GR dependent, is also less efficient in the absence of GerP proteins (69). In contrast, germination induced by HHP, which is thought to directly activate GRs or SpoVA channels, is unaffected by *gerP* deletion (69). Interestingly, germination induced by the non-GR dependent germinant dodecylamine is more rapid in *gerP* deletion mutant compared to the wild type (WT), although the reason remains unclear (42). It is postulated that the GerP proteins may play a protective and selective role in the spore’s outer layer, allowing the entry of nutrient germinants or Ca-DPA that signifies favorable conditions, while preventing the sporicidal agents such as dodecylamine that indicates the adverse conditions (76–78).

### GerA

In *B. subtilis*, GerA is the most extensively studied GR. GerA homologs are widely distributed in spore-forming bacterial species, and hence termed GerA-type GRs (28, 79, 80). These GRs normally consist of three subunits A, B, and C, and sometimes with a D subunit, located within the IM of spore (81, 82). Significantly, all three subunits play a crucial role in maintaining the function of the GRs, as the absence of any single one affects the GRs’ complex assembly and thus renders them non-functional (83, 84). The primary function of GerA is to receive and transduce the germination signal by binding with either l-Ala or l-Val (85, 86). In addition, GerA also plays a crucial role in the response to HHP-induced germination (67). It was recently revealed that GerA consists of a complex of proteins that form a nutrient-gated ion channel in the spore IM, though the number of subunits assembling the channel is controversial (31, 87). The research by Gao et al. presents intricate details regarding the structure of GerA complex (31). Their study reveals that GerA, predicted by Alphafold2-multimer, is a sophisticated pentameric complex of GerAA-GerAB-GerAC trimers (31). However, Kilian and Bischofs also employ Alphafold2-multimer and co-evolution analysis, revealing that GerA is more likely a hexamer, which has slightly more native, co-evolving intermolecular contacts than pentamer (87). More detailed experiments, including co-immunoprecipitation coupled with pull-down and mass spectrometry, as well as cryoEM, should identify the exact composition of GerA. Nonetheless, helix three of the GerAA subunit is predicted to form the narrowest section of ion channel, which traverses the entire spore IM and extends into the spore core (31). The presence of acidic residues around the channel suggests its selectivity for cations and its role in controlling ion release. The GerAA pentameric or hexameric structure is surrounded by GerAB subunits within the membrane, and both GerAA and GerAB interact with GerAC, which is located outside the membrane (31).

According to Gao et al., the activation of GerA by l-Ala may proceed as follows (31, 55): l-Ala first binds to the binding pockets on the GerAB subunits, changing their structure. This change induces further structural adjustments in GerAA through the interaction between GerAB and GerAA subunits, leading to GerA channel opening and ion release. Subsequently, the opening of the channel and ion release trigger SpoVA channel opening, resulting in a significant release of Ca-DPA. However, the precise role of ion release remains unclear. Recent research indicates that the release of K^+^, regulated by the potassium channel protein YugO, initiates subsequent germination events by generating a membrane potential, as detected through thioflavin-T accumulation (33). However, Li et al. showed that the thioflavin-T accumulation during the early stages of germination results from its binding to spore coat proteins, rather than changes in the electrochemical potential of spore membrane (34). This discrepancy highlights the ongoing debate regarding the role of ion release in activating downstream germination events and underscores the need for further investigation.

### GerB/GerK

GerB and GerK are homologous to GerA, with each also consisting of A, B, and C subunits (24, 25, 28, 88). While the structure of GerA has been relatively detailed studied by bioinformatics and biochemical analyses, the structures of GerB and GerK are only predicted (31, 89). GerB and GerK play crucial roles in AGFK-induced germination, but overexpressing these receptors does not increase the AGFK germination rate (90). Interestingly, overexpression of GerB enables spores to respond to l-Asp and d-Ala, thereby inducing germination (90). Studies suggest that GerB has a relatively nonspecific l-amino acid binding site, but it is typically hidden (86). Hence, though GerB can bind to l-amino acids and elicit a response, these amino acids typically have limited access to the binding site. As a result, GerB is unable to initiate germination on its own (25, 86). For GerB to effectively respond to l-amino acids, it must interact with GerK and GFK germinants (86). The presence of GerK and GFK germinants likely induces structural alterations in GerB, exposing its binding site and allowing GerB to respond to various amino acids, which then triggers germination (86). As for GerK, its precise function is not entirely clear. Studies suggest that GerK promotes germination in conjunction with GerB and GFK (86). Some researchers indicate that GerK primarily responds to glucose, but further investigation is needed to confirm its exact mechanism of action (91). In addition to respond to nutrient germinants, GerB and GerK can also respond to 150 MPa pressure, with their sensitivity ranked as GerA > GerB > GerK. Germination induced by 550 MPa, Ca-DPA or dodecylamine is not affected by GerB and GerK, probably because germination induced by these factors occurs independently of GRs (92, 93).

### GerD

In *Bacillus* species, GerD is a lipoprotein located on the outer surface of the spore IM, and it is expressed in the developing forespore compartment of the sporulating cell concurrently with *gerA*, *gerB*, and *gerK* operons (72). GerD forms a stable α-helical trimer in aqueous solutions, with a unique and complex structure resolved at 2.3 Å resolution, resembling a twisted superhelical rope made of intertwined hydrophobic screws from interacting helices (94). This special structural arrangement of the GerD trimer, characterized by its intricate design, is believed to play a crucial role in clustering of GRs within the IM of dormant spores, thereby promoting a rapid and cooperative response to germinants (94). Consequently, the GerD is crucial for the proper function of GRs, as Δ*gerD* mutant spores display a 10- to 20-fold decrease in germination rate with nutrient germinants like l-Ala or AGFK, and this is particularly obvious under 150 MPa that activate spore germination through GRs (20, 72, 95, 96). However, the absence of GerD does not affect germination induced by non-nutrient germinants such as a Ca-DPA, dodecylamine, lysozyme, or pressure of 500 MPa in spores lacking all nutrient receptors. This highlights GerD’s specific role in rapid responses to nutrient germinants (72). However, the specific mechanism of how GerD clusters GRs, needs further investigation.

### GerF

In *B. subtilis*, GerF is the sole prelipoprotein diacylglycerol transferase (74, 97), with the primary function of catalyzing the transfer of diacylglycerol to specific Cys residues in membrane prelipoproteins (74). The deletion of *gerF* can cause defects and slow down the process of spore germination induced by nutrient germinants (73, 98). However, this deletion has no effect on spore germination induced by Ca-DPA or dodecylamine (73). Generally, the A and B subunits of GRs are hydrophobic and contain several transmembrane regions, while the C subunit and GerD are hydrophilic and anchored on the outer surface of spore IM (25, 99). Studies have shown that the C subunit and GerD possess an N-terminal signal sequence with a consensus sequence for diacylglycerol addition at a specific Cys residue (97, 100, 101). This diacylglycerol addition presumably prevents signal peptidase II from cleaving the signal peptide, thereby ensuring the proper localization and normal function of these hydrophilic proteins (102). Therefore, diacylglycerol addition is crucial for GR’s assembly and ensures their normal function. Interestingly, despite possessing the same Cys residue for GerF recognition, GerK appears to be less affected by either the absence of GerF or mutation of its Cys residue (73). This resilience may be because GerKC has a lengthy hydrophobic region preceding the diacylglycerylated Cys, making it unlikely to be removed by signal peptidase II (97, 102). However, the specific mechanism of GerF action remains to be explored.

### YwlE/SigA/Tig/RpmE

Protein phosphorylation, a widespread mechanism facilitating rapid cellular reactions to external signals, is considered to be partially involved in germination signal transduction (103, 104). In *B. subtilis*, McsB is the kinase responsible for Arg phosphorylation, counterbalancing the action of the Arg phosphatase YwlE (105–107). Recently, Zhou et al. found that Δ*ywlE* spores exhibit significant germination deficiencies, along with the slower Ca-DPA release and heat resistance loss compared to WT spores, while Δ*mcsB* spores germinate faster than WT spores (44). These results suggest that the Arg phosphorylation of some essential spore proteins is executed by McsB during sporulation; when the dormant spores are stimulated by germinants, these phosphorylated sites are removed by the YwlE to propel germination.

Further research demonstrated that YwlE is essential for the dephosphorylation of Arg365 and Arg45 residue in housekeeping sigma factor SigA and translation factor Tig, respectively, thereby activating transcription and translation (44). In dormant spores, deletion of *sigA* hinders transcriptional activity, leading to noticeable germination defects (22, 44). The dephosphorylation of Arg365 in SigA facilitates its binding with DNA and is integral in initiating transcription, indicating that YwlE-mediated dephosphorylation of SigA plays an important role in spore germination (44). Tig is a ribosomal-associated molecular chaperone that regulates translation by directly interacting with newly synthesized polypeptides to assist in the folding of cytosolic proteins (108). Dephosphorylation of Tig Arg45 by YwlE is required for Tig to promote translation and facilitate germination events (44, 45). RpmE, a non-essential component of the 50S ribosomal subunit, is also important for promoting germination (44, 45). According to these above findings, it appears that the synthesis of macromolecules through transcription and translation occurs early in germinating spores and regulates the germination process. However, as mentioned above, this new point of view is still largely controversial (also see the “Perspective” section) (42, 46–49), largely due to the lack of adequate amount of ATP to energize these metabolic activities (41, 109, 110). Researchers even find that spores lacking the crucial energy source 3-phosphoglycerate or translational machinery rRNAs, germinate normally though the spores accumulated no detectable ATP (46, 111). Currently, we do not have a reasonable explanation for this controversy. By utilizing new technologies such as single-cell transcriptomics, proteomics, or metabolomics, perhaps we can more accurately reveal the metabolic activity status of spores at the early stages of germination. In this way, the controversy may be resolved, and the regulatory mechanism for the early germination events will become clearer.

### SpoVA

In *B. subtilis*, the SpoVA proteins are encoded by *spoVA* operon consisting of seven genes, including *spoVAA*, *spoVAB*, *spoVAC*, *spoVAD*, *spoVAEb*, *spoVAEa*, and *spoVAF*. These proteins are expressed in the forespore in parallel with GRs and GerD genes, and localized to the spore IM, functioning as a channel for Ca-DPA transport during sporulation and germination (25, 28, 38, 61, 70, 112, 113). It is reported that any gene deletion within the *spoVA* operon, excluding *spoVEa* and *spoVAF*, impairs the Ca-DPA transport function of SpoVA during sporulation (70), while overexpression of *spoVA* operon can accelerate the rate of Ca-DPA release during spore germination (38). Further detailed studies on each SpoVA subunit indicate that SpoVAC, SpoVAD, and SpoVAEb form the minimal Ca-DPA transport channel, which is critical for Ca-DPA accumulation during sporulation and release during germination (70). Specifically, SpoVAC and SpoVAEb form the membrane channel, whereas SpoVAD assumes a plug-like function in the cytoplasm (20, 70). However, earlier studies indicate that SpoVAD is likely anchored to the outer surface of the spore IM (114), where it binds and transports Ca-DPA into developing spores (42). The deletion of either SpoVAA or SpoVAB leads to premature spore germination, while the deletion of *gerA* can suppress this phenotype and generate spores with normal Ca-DPA level (70). Hence, these two proteins are likely involved in assisting the SpVAC-D-Eb channel in releasing Ca-DPA during germination (70, 87). SpoVAEa and SpoVAF do not participate in Ca-DPA accumulation during sporulation, but they impact Ca-DPA release during germination (115). Recent studies have revealed that SpoVAEa moves within the IM randomly with high frequency, potentially communicating signals from GRs to SpoVA channel, thereby facilitating Ca-DPA release (116, 117). A recent study by Gao et al. indicates that SpoVAF, together with its partner protein YqhR (renamed as FigP), assemble into an oligomeric ion channel. This channel acts as a signal amplifier to enhance the germination rate by accelerating ion release together with GerA in the early stage of germination (32). In summary, SpoVAC-D-Eb forms a Ca-DPA transport channel, with SpoVAA, SpoVAB, and SpoVAEa playing roles in its normal function. SpoVAF/FigP form an ion channel complex that increases ion release efficiency, thereby facilitating Ca-DPA release and the subsequent germination process. However, how ion release regulates Ca-DPA release is not clear, and needs to be further studied.

### CwlJ/SleB

CwlJ exhibits lytic transglycosylase catalytic activity and is thought to reside in the inner spore coat adjacent to the outer surface of the cortex, with the assistance of SafA, CotE, and GerQ (35, 42, 53). Similarly, SleB is also a lytic transglycosylase located in the spore IM and the inner coat-cortex region, respectively, adjacent to the cortex inner and outer surfaces with the assistance of YpeB (50). In *B. subtilis*, CwlJ and SleB are synthesized in mature form in the mother cell and forespore, respectively (50, 118), but how they remain inactive in dormant spores is unclear. The main function of CwlJ/SleB is to hydrolyze the cortex while keeping the germ cell wall integral during spore germination, as they can exclusively recognize the muramic-δ-lactam (MAL) modification in cortex PG (50). Either CwlJ or SleB is sufficient to mediate cortex hydrolysis, but the loss of both enzymes prevents cortex degradation and thus germination (50, 54, 119). Interestingly, Δ*cwlJ* spores exhibit significantly slower Ca-DPA release during GR-mediate germination in contrast with their behavior when germination is induced independent of the GRs (20, 119). However, this phenotype is not present in Δ*sleB* spores (119). These results indicate that the CwlJ-induced cortex hydrolysis may have specific effects on the Ca-DPA channel different from those of SleB.

It has been proposed that CwlJ is activated by Ca-DPA released from spore core during early germination stages, or the external high-concentration Ca-DPA (60, 119), but the exact mechanism is unclear. Recently, Amon et al. found that SwsB, broadly conserved among spore formers, is required for CwlJ to efficiently degrade the cortex during germination (53). Since SwsB resembles polysaccharide deacetylases, and its putative catalytic residues are required for its role in germination, the authors propose that SwsB modestly modifies the cortex to increase the degradation efficiency of CwlJ (53). In contrast, SleB cannot be activated by either endogenous or extracellular Ca-DPA (60). Instead, its activation is thought to be mediated by the structural changes of the cortex PG caused by partial core hydration during the early stages of germination (25). Moreover, SleB’s partner protein YpeB may also be involved in regulating its activity (50). Nonetheless, while the roles of CwlJ and SleB during germination appear quite clear, the mechanisms by which they are activated need to be further studied.

### Newly identified spore IM proteins

The YlaJ, YhcN, YutC, and CoxA all belong to spore lipoprotein family, and are highly expressed in developing forespore (120, 121). A recent study found that the absence of one or more of these lipoproteins will lead to significant spore germination defects induced by l-Ala (122). Specifically, spores lacking these proteins show slower Ca-DPA release, loss of heat resistance and decreased optical density (OD) during germination (121). However, these effects are not observed in AGFK-induced germination, which may be attributed to the inherently slower germination efficiency of AGFK, possibly masking the effects (123). Based on their further study, the authors propose the following interpretations of how these lipoproteins affect germination: (i) these lipoproteins, especially YlaJ and YhcN, enhance the fluidity of the spore IM (121); (ii) they may interact with other germination-related proteins, such as GRs, to maintain them in a relatively discrete environment, thus ensuring their normal function (121).

Moreover, according to the recent work of Yu et al., there are another two hydrophobic proteins YetF and YdfS identified in the spore IM, also involved in spore germination and resistance (124). These two proteins are exclusively identified in *B. subtilis* spores and are believed to be situated in the IM of spores (124, 125). Notably, YetF or YdfS deficient spores show severe GRs-dependent germination defects, along with less resistance to DNA-damaging chemicals and wet heat (124). It is hypothesized that YetF and YdfS may modify spore IM permeability by tweaking its structure, which likely stabilizes the IM proteins, such as GRs and/or the germinosome, to ensure their normal function (124). Additionally, YetF and YdfS are homologous to 2Duf, an IM protein with two transmembrane domains (Duf421 and Duf1657), which is believed to be the chief contributor of resistance to wet heat (126, 127). However, the precise mechanisms behind these lipoproteins are yet to be fully understood.

## THE PROTEINS REGULATING SPORE QUALITY

**Fig 2:**
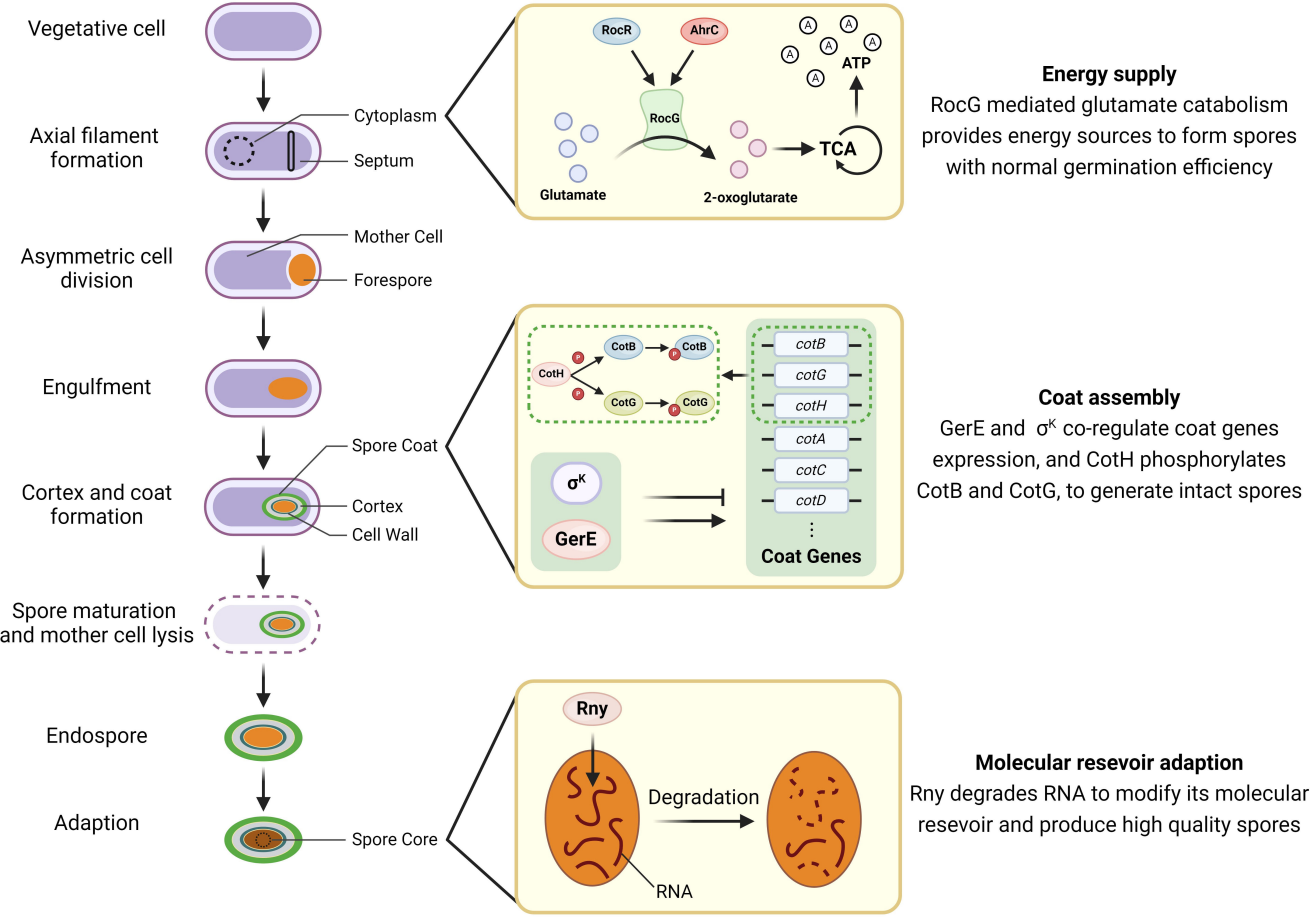
The proteins regulating the sporulation of *Bacillus subtilis* to affect future spore germination. Sporulation commences with the asymmetric division of a sporangium, resulting in the creation of two separate compartments, namely the mother cell and the forespore. Following this, the mother cell proceeds to engulf the forespore, resulting in the formation of a forespore encased in a double membrane. Then, the cortex is synthesized between the two membranes, and the coat is assembled outside the outer membrane. Subsequently, the spore continues maturation with Ca-DPA accumulation and core dehydration, followed by the mother cell lysis. Finally, the released spore undergoes adaptive period to enhance survival ability. At the onset of sporulation, RocG-mediated glutamate catabolism provides energy sources to form spores with normal germination efficiency; At the stage of structure assembly, GerE and σ^K^ co-regulate coat gene expression, and CotH phosphorylates CotB and CotG, to generate intact spores. At the adaptive period, RNase Y (Rny) degrades RNA to modify its molecular reservoir, producing high-quality spores to survive diverse environments.

The sporulation process greatly affects the quality of spores, thereby influencing their subsequent germination and outgrowth (128). Spores developed in nutrient-rich media possess higher levels of GRs compared to those formed in nutrient-deficient environments, resulting in a more effective response to germination stimuli (25, 66, 90). Sporulation temperature also markedly affects the germination of *B. subtilis* spores. Specifically, spores formed at higher temperatures (above 37°C) show reduced germination efficiency with l-Val/AGFK, high hydrostatic pressure (500 MPa), or dodecylamine, compared to those produced at lower temperatures (below 30°C) (68, 93, 128, 129). Apart from external factors, proteins produced during sporulation that are not directly involved in the germination process can nevertheless influence the quality of spores. These proteins include GerE, a DNA-binding protein that affects the expression of numerous spore coat proteins (26, 130); RocG/RocR/AhrC, which are involved in arginine and glutamate metabolism (27); Rny, which is important for the formation of RNA degradosome (131); and CotH, protein kinase involved in phosphorylation (132, 133). Subsequent sections will provide detailed introductions to each protein (Fig. 2).

### RocG

RocG is a glutamate dehydrogenase in *B. subtilis*, and its expression is regulated by the transcription factors RocR and AhrC (27, 134, 135). Recent studies find that the deletion of *rocG* significantly reduces sporulation efficiency, while the deletion of both *rocR* and *ahrC* has no effect on sporulation but instead results in severe germination defect (27). Meanwhile, *rocG* overexpression leads to adverse effects on future spore properties, for example, increased germination efficiency, reduced DPA content, and lowered heat resistance (135). These results suggest that glutamate metabolism during sporulation greatly affects this process and the future spores’ properties (27, 135). Based on their research, the authors propose that the RocG-mediated glutamate catabolism at the beginning of sporulation, generates adequate amount of ATP for energizing sporulation completion as well as ensuring high spore quality for normal germination (27, 135). Moreover, the RocG product 2-oxoglutarate also has an anabolic function acting as a precursor for the synthesis of amino acids and nucleotides, as well as contributing to the NADH pool (136). In this way, it can also affect the spore molecular content or structure via alternative metabolic pathways, and thus affect spore germination. However, how ATP or the potential molecular content participates in regulating spores’ properties has yet been established.

### GerE

GerE, as a transcription factor, modulates late-stage sporulation by regulating the expression of genes encoding spore coat proteins (137). The expression of *gerE* is regulated by the σ^K^ transcription factor, and they can subsequently cooperate to induce (*cotC*, *cotD*, *cotG*, *cotQ*, *cotS*, *cotU*, *cotV*, *cotW*, *cotX*, *cotY*, and *cotZ*) or repress (*cotA*, *cotB*, *cotE*, *cotH*, *cotM*, *cotT*, and *cotP*) the expression of coat protein-encoding genes during the final stages of coat assembly and spore maturation (26, 130, 137, 138). Therefore, the deletion of *gerE* can induce the absence of the spore outer coat and alternations in the inner coat layers (139). Sturm and Dworkin found that Δ*gerE* spores exhibit elevated spontaneous germination—a bet-hedging strategy for survival—though these spores exhibit notable impairments in nutritional germination (130, 140). Specifically, spores lack GerE display higher susceptibility to lysozyme, which may result from the absence of an inner coat (26). This enables the spores to more easily sense the external environment, thus exhibiting a higher rate of spontaneous germination (26). In contrast, spores with high levels of GerE may show inhibition in spontaneous germination, suggesting that the spores lose partial ability to respond to environmental changes (26). Based on these results, the authors propose that the variation of GerE level in individual sporulating cells leads to corresponding spores possessing different extents of coat defects, which determines each spore’s spontaneous germination capability (26). Hence, GerE likely exerts genetic control over spore quality via coat modification, regulating spontaneous germination and benefiting spore survival.

### CotH

CotH, initially believed to be a *B. subtilis* spore coat protein regulated by σ^K^, plays a key role in assembling various outer coat components and conferring lysozyme resistance to mature spores (141, 142). It has been long for researchers to observe that the CotH-deficient spores exhibit significant reduction of germination efficiency induced by l-Ala (141). Recently, Nguyen et al. identified CotH as a protein kinase, and CotH-dependent phosphorylation of CotB and CotG is required for the efficient spore germination (132, 133). In particular, CotH induces multisite phosphorylation of CotB during sporulation, facilitating the conversion of CotB immature form CotB_46_ into its mature form CotB_66_ (132, 133). Meanwhile, CotH-mediated phosphorylation of CotG protects it from being hydrolyzed. These effects are crucial for the proper assembly of proteins into the spore coat, and ultimately facilitate efficient germination.

### Rny (RNaseY)

The endoribonuclease RNaseY is encoded by single-gene operon *rny*, and plays a key role in forming the RNA degradosome involved in mRNA processing in *B. subtilis* (143). This enzyme comprises an N-terminal transmembrane domain, an RNA-binding domain, and a C-terminal domain involved in metal-dependent phosphohydrolysis (143, 144). RNaseY initiates degradation specifically within certain mRNA regions, such as the single-stranded sequence six to nine nucleotides upstream of the thrS leader terminator structure (145). It has been reported that the deletion of the *rny* gene severely impairs sporulation efficiency (146). However, by using an inducible *rny* expression strain, Segev et al. have revealed that RNaseY-depleted strain produces spores with significant delays in germination and outgrowth (131). Meanwhile, these RNaseY-depleted spores display reduced RNA degradation levels, indicating that the RNA content within dormant spores influences their germination capability (131). Their further research indicates that RNaseY modulates the RNA levels in spores during the interval between the release of the mature spore from the mother cell and the achievement of dormancy (131). This modulation is believed to enhance the adaptability of bacterial spores to various natural environments for survival.

## PERSPECTIVE

Bacterial spores have gained significant attention due to their threat to the food industry and human health. Spores in a dormant state have no danger, but they can rapidly resume metabolic activity to cause adverse effects once being triggered to germination. Thus, understanding the mechanism of the germination process is of great significance to develop new strategies for eliminating the risks brought by spores. By using *B. subtilis* as model organism, current research on spore germination has detailed its main physiological events and identified many of the proteins required for these events, including GRs for signal transduction, the SpoVA channel proteins for Ca-DPA release, and CLEs CwlJ and SleB for cortex degradation. Moreover, accessory proteins have also been identified such as GerD for clustering the GRs, IM proteins (YlaJ, YhcN, YutC, CoxA, YetF, and YdfS) for stabilizing GRs, and YpeB and SwsB for regulating CLE activity. Excitingly, GerA has been revealed to be a nutrient-gated ion channel, synergistically working with SpoVAF/FigP complex, which releases ions in the early stages of germination (32). In addition, transcription and translation may occur during these early germination events, which are earlier than previously assumed. Thus, research on spore germination has made considerable progress in recent years. However, as alluded to throughout this review, there are still several key issues to be addressed in future research.

1. What is the exact mechanism of GerA activation? Though GerA’s function as an ion channel has been established, how this channel opens after germinants binding is unclear. To solve this problem, molecular dynamics simulations during germinant binding can be employed to predict the channel opening model of GerA, which can be further verified by genetic manipulation. Alternatively, cryo-EM is also a promising approach. Another important question is whether other GerA-type GRs, for example, GerB, GerK, and even homologs in other spore-forming bacteria, also ion channels and play the similar roles? To investigate this, these homologous can be artificially expressed and studied in the background of *B. subtilis* strain without any GerA-type GRs.
2. What ions are released from GerA and other potential channels and what is the function of this event? Since Ger receptor activation is needed for Ca-DPA release, ions released from the Ger complex must directly or indirectly induce the release of Ca-DPA. Indeed, latest research provides evidence supporting that activation of SpoVA by GerA is not mediated by protein-protein interactions and instead involves some chemical or physical change to the spore (31), signifying that GerA-mediated ion release may indirectly lead to Ca-DPA release. However, more detailed experiments such as dynamic stimulation combined with gene manipulation are needed to provide more evidences to reveal the underlying mechanism.
3. Do spores indeed reactivate metabolic processes in the early stages of germination? Since transcription and translation appear to be detected when germinating spores are still phase-bright (22, 43–45), a stage prior to or at when Ca-DPA release occurs, the possibility cannot be dismissed. However, this perspective remains a contentious issue, primarily due to the extremely low ATP levels in dormant spores (41, 109, 147), which are insufficient to drive transcription or translation. Although research suggests that malate can serve as a significant energy source for ATP generation (45), this has also been challenged, mainly based on the detection of only trace amounts of malate in the dormant spore core (47, 109). To resolve this debate, two avenues of study could be pursued: (i) *In situ* experiments, such as using time-lapse microscopy and fluorescence labeling to examine the metabolic activities and Ca-DPA release of individual spores, are needed to establish the uncertain “cause and effect” relationship between metabolic activities and Ca-DPA release. (ii) Single-cell multi-omics technologies can be employed to detect the metabolic activities within a single dormant or germinating spore, thereby accurately delineating the timeline of spore revival.
4. Are there any more proteins involved in germination yet to be discovered? As new germination-related proteins have continuously been identified in the recent few years, the answer to this question appears to be yes. Fortunately, the successful construction of whole-genome scale genes knock-out *B. subtilis* strains libraries (148, 149), along with the development of the efficient TnSeq screen method (150), make the thoroughy screening of new genes regulating germination become achievable. Identification of these undiscovered germination proteins can possibly solve the questions mentioned above, thus promoting our understanding of germination.
